# Comparison of the IOLMaster 700 and the Pentacam in the Analysis of the Lens Nuclear Density Before the Cataract Surgery

**DOI:** 10.3389/fmed.2021.691173

**Published:** 2021-10-20

**Authors:** Bowen Li, Yuqi Liu, Yiping Hu, Mingyu Shi

**Affiliations:** ^1^The Department of Ophthalmology, The Fourth People's Hospital of Shenyang, Shenyang, China; ^2^The Department of Ophthalmology, The Fourth Affiliated Hospital of China Medical University, Shenyang, China; ^3^The Department of Ophthalmology, China Medical University, Shenyang, China

**Keywords:** cataract, nuclear density, swept-source biometer, Scheimpflug system, IOLMaster 700

## Abstract

**Purpose:** To evaluate the difference of the lens nuclear density measured before and after mydriasis by using the IOLMaster 700 (Carl Zeiss Meditec AG, Jena, Germany) and the Pentacam Scheimpflug imaging (Pentacam HR, Oculus Incorporation, Wetzlar, Germany) and investigate the relationship between the measurement data and the phacoemulsification parameters.

**Methods:** Patients with age-related nuclear cataracts diagnosed on the slit-lamp examination were enrolled in the age range of 53–76 years. No patient had a history of ocular surgery, laser treatment, or general disorders affecting vision. The mean optical density (OD) was measured by the IOLMaster 700 by using the Image-Pro^®^ Plus software before and after mydriasis. The Pentacam nucleus densitometry (PND) was obtained automatically from the Pentacam Scheimpflug imaging and compared with OD. The correlation between OD and effective phacoemulsification time (EPT), PND, and EPT were analyzed, respectively.

**Results:** In this study, 53 eyes of 52 patients were evaluated. Before and after mydriasis, the mean OD values were 64.34 ± 23.31 and 63.81 ± 23.21 pixel units, respectively; the mean PND values were 28.51 ± 11.42 and 25.41 ± 11.31, respectively; and the mean EPT value was 6.24 ± 3.49. The Bland–Altman analysis showed that the lens nuclear densities of the two devices were highly consistent. There was no significant difference in the OD values (*t* = 0.455, *p* > 0.05) before and after mydriasis, but the difference has existed in the PND values (*t* = 2.509, *p* < 0.05). The OD and PND values were positively correlated with EPT before and after mydriasis (*r*_OD−Before_ = 0.604, *r*_OD−After_ = 0.593, *r*_PND−Before_ = 0.701, and *r*_PND−After_ = 0.891, *p* < 0.01).

**Conclusion:** The combination of the IOLMaster 700 and the Image-Pro^®^ Plus software can quantitatively evaluate the degree of the cataract lens opacification. It has good consistency with the Pentacam and is positively correlated with the phacoemulsification parameters. It is expected to become a new method to predict the phacoemulsification parameters before and during cataract surgery.

## Introduction

A cataract is a common public health issue and the major cause of blindness in the world (1). Quantitative measurement of the lens density of cataracts is vital in evaluating the possible risk factors and optimizing the therapeutic schedule. The Lens Opacities Classification System III (LOCSIII), a subjective method of assessing cataracts, is considered as the standard classification system for evaluating the cataract morphology and opacification (2–4). However, the practical application of thesubjective methods is limited by the observer bias and repeatability of measurements to some extent (5). Therefore, the objective quantitative lens density measuring is of great significance for evaluating the possible treatment risk factors and optimizing treatment plans. Moreover, in some cases of cataract surgery, such as cataracts in the post-keratotomy and in keratoconus, the intraoperative ultrasound energy may cause the dehiscence in the radial scars (6). Thus, it can be seen that the measurement of the lens nucleus density before cataract surgery is particularly important. Intraoperative phacoemulsification energy can be estimated by measuring the lens density of the cataract before the operation and then the corneal condition after the operation can be inferred.

The Scheimpflug tomography (Pentacam HR, Oculus Incorporation, Wetzlar, Germany) has been proved to be a reliable objective method for classifying the lens density (7–10). It is non-contact diagnostic imaging equipment for ophthalmology based on the Scheimpflug optical principle. The instrument sets a 50-scan acquisition mode to capture 25,000 elevation data points in 2 s. Operators can achieve a three-dimensional image of the whole lens in real-time (11, 12). Lens density can be measured accurately by the Pentacam after mydriasis (13). However, the Pentacam measurement is limited by many external factors, such as the excessive lens opacity will lead to light blocking, which will affect the accuracy of the measurement results (14).

In addition to the optical biometry, swept-source optical coherence tomography (SS-OCT) technology is based on time-varying spectral interferometry (15). In short, the wavelength scanning laser source is divided into the sample arm and the reference arm. A-scan is obtained by the Fourier transform of the interference signal and provides the reflectivity profile on uniaxial. A plurality of the individual A-scans is combined laterally to generate a cross-sectional image reconstruction forming a B-scan. A three-dimensional reconstruction can be obtained by acquiring B-scans at the different continuous positions. The high acquisition speed of the swept-source device allows multiple scans at the same location, which can improve the image quality through image averaging (16). Because of its imaging principle, it is not only used in the diagnosis of the retinal diseases, but also in the research of the cataract. Brás et al. have reported that the SS-OCT measurements allowed the quantification and documentation of the cataract density (17). The IOLMaster 700 (Carl Zeiss Meditec AG, Jena, Germany), a high-precision instrument also based on the SS-OCT principle, shows the vertical structure of the entire eye and its center wavelength of the light source is 1,055 nm, which scans from the six directions. It provides a full-range OCT mode and can clearly display the cornea, iris, lens, and the other anterior segments of the ocular structures. Therefore, it is possible to reflect the opacity of the cataract lens intuitively.

The purpose of this study was to determine a new objective method for grading the cataract severity by using the average lens nuclear density measured by IOLMaster 700 and to evaluate the correlation between the lens density and the phacoemulsification parameters for the age-related nuclear cataract compared with the Pentacam before and after the pupil dilation. This study shows the comparison of the lens density measured by the two optical devices before and after mydriasis and the correlation with the energy parameters of phacoemulsification.

## Materials and Methods

### Patient Selection

Subjects were recruited from August 2019 to January 2020 at the Fourth Affiliated Hospital of China Medical University. About 52 patients (53 eyes) with the age-related nuclear cataract diagnosed on the slit-lamp examination were enrolled in the age range of 53–76 years. No patient had a history of ocular surgery, laser treatment, or general disorders affecting vision. After receiving full information of the details and possible risks of the study, all the patients provided informed consent. The study was performed in accordance with the tenets of the Declaration of Helsinki and approved by The Ethics Committee of the Fourth Affiliated Hospital of China Medical University and registered at www.chictr.org.cn. (study registration no. ChiCTR2000029380).

### Swept-Source Optical Coherence Tomography Measurements and the Scheimpflug Measurements

**Figure 1:**
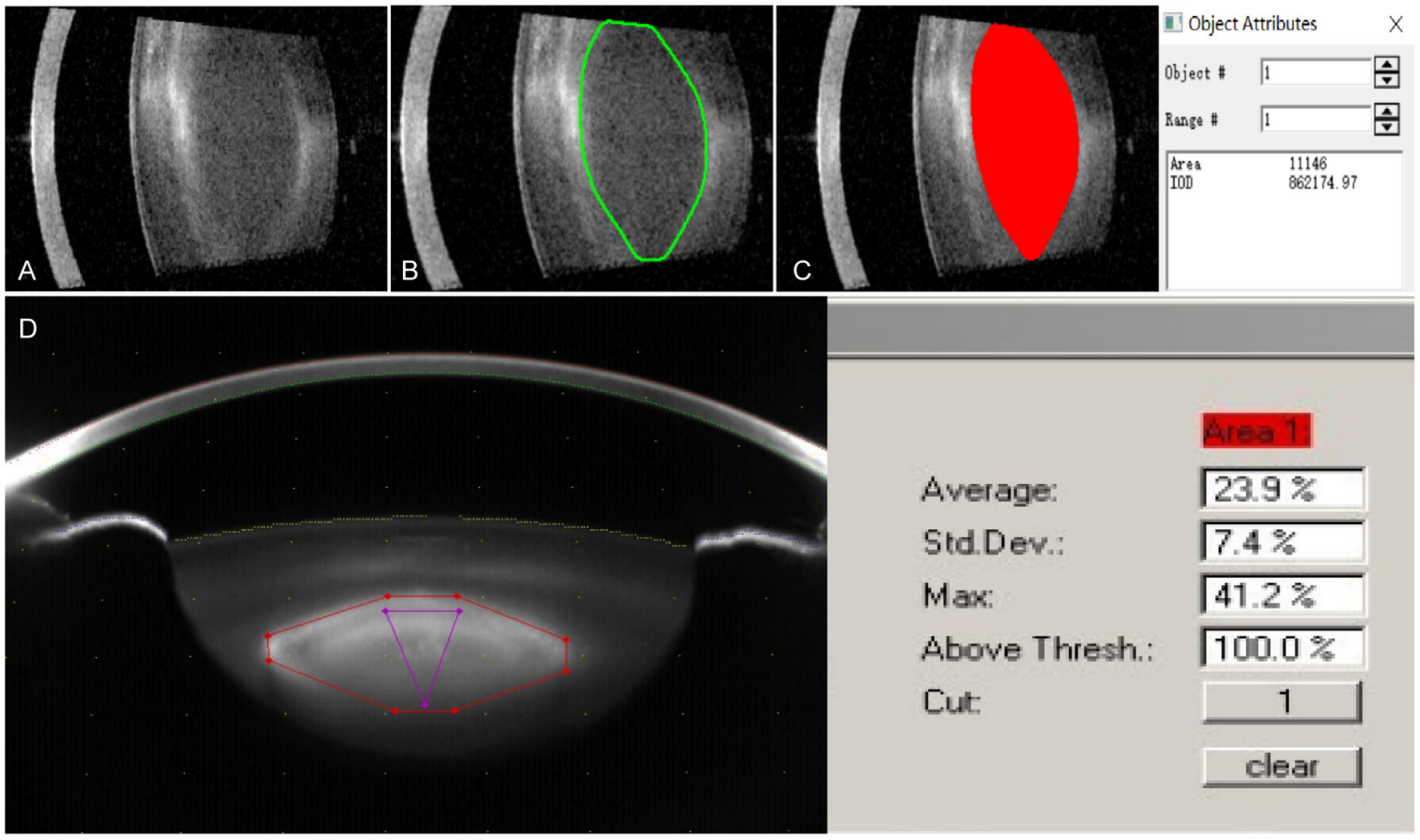
The lens image of the IOLMaster 700 was exported to the Image-Pro^®^ Plus software for measuring the lens opacity degree in the region of interest indicated by the red area. **(A)** The lens images obtained by the IOLMaster 700; **(B)** Selected lens nucleus area of the IOLMaster 700; **(C)** The optical density (OD) value of the lens nucleus density was analyzed by Image-Pro^®^ Plus software; and **(D)** The lens image was obtained by the Pentacam.

All the subjects were performed the IOLMaster 700 (Carl Zeiss Meditec AG, Jena, Germany) and the Scheimpflug system (Pentacam HR Oculus Incorporation, Wetzlar, Germany) examinations before and after mydriasis and the clear lens images of these two optical devices were captured by the same examiner, respectively. It has been confirmed that some areas of the lens are more representative of the opacity and the changes in the severity of nuclear sclerosis (or lens density) compared to others (5, 10). So, we defined a standard rectangular region of interest (ROI) that excluded the lens cortex and the mean optical density (OD) of ROI means the lens nuclear density with the IOLMaster 700. After exporting the two-dimensional image data captured with the SS-OCT of the IOLMaster 700 to the Image-Pro^®^ Plus software (Media Cybernetics, Inc.), the OD value was measured in the pixel units of intensity in the range of 0–255. We selected ROI with high precision and then made a concrete analysis (Figures 1A–C). This method was repeated three times for all the automatic six angles acquisition of each eye and the average lens nuclear density was calculated. The three-dimensional lens images acquired by the Pentacam consistent with the six directions of the IOLMaster 700 were selected to analyze the average lens nuclear density and quantified on a scale of 0 to 100 (0 = no cloudiness; 100 = completely opaque lens). Through the image analysis software of the Pentacam, the lens nucleus area was selected for analysis and the system automatically generated the Pentacam nucleus densitometry (PND) value (Figure 1D).

### Phacoemulsification and Effective Phacoemulsification Time

All the phacoemulsifications with the same operation parameters were performed in the capsular bag by using the Stellaris System (Bausch & Lomb^®^ Laboratories) by a single experienced surgeon. The routine stop-and-chop quadrant-aspiration technology and linear mode were selected; the height of the perfusion bottle was 90 cm; the cortical suction negative pressure was 450 mm Hg, the upper energy limit was 100%, the flow rate was 45 ml/min; and the cortical suction negative pressure was 600 mm Hg and the flow rate was 40 ml/min. Balanced salt solution (BSS plus; Alcon^®^ Laboratories) was used as an irrigation solution. At the end of each surgery, the mean ultrasound energy and torsional time were recorded automatically on the screen and the effective phacoemulsification time (EPT) was also calculated (mean ultrasound energy × torsional time/100%) (9).

### Statistical Analysis

The statistical analysis was performed by using GraphPad Prism 7.0. The sample size was calculated as follows: *n* = *Z*^2^ × δ^2^/*e*^2^ [*Z* (CI) = 1.96; δ (SD) = *p* (1-*p*), *p* = 0.5; *e* (sampling error range) = 10%; *n*, sample size]. As the sample size exceeds the overall capacity by 5%, the sample size shall be adjusted [*n*' = (*n* + *N*)/(*n* + *N*-1) (*n*, original sample size; *N*, overall capacity; *n*', adjusted sample size)]. The Kolmogorov–Smirnov test was used to test whether the measured data were normally distributed. The results showed that the data were in accordance with the normal distribution. The paired *t*-test was used to evaluate the difference between the OD and PND values before and after mydriasis. The relationship between the OD value and PND value was analyzed by using the Bland–Altman plots. In addition, the Pearson correlation analysis was performed to evaluate the relationship between the OD value and EPT value and the PND value and EPT value. *p* < 0.05 was considered as statistically significant.

## Results

**Figure 2:**
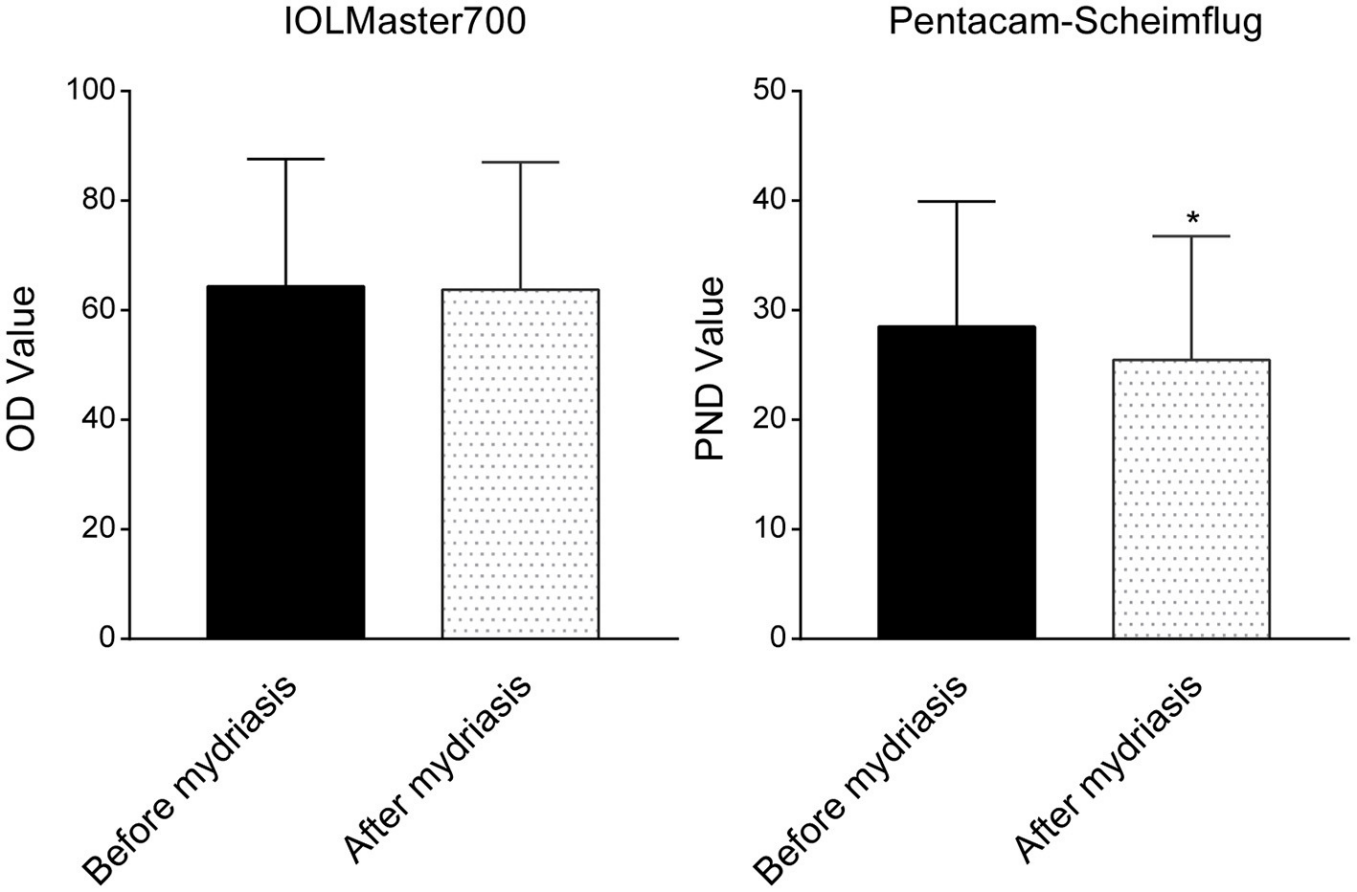
Comparison of the lens density before and after mydriasis obtained with the IOLMaster 700 and the Pentacam Scheimpflug imaging. There was no significant difference between the two groups (*t* = 0.455, *p* > 0.05) in the IOLMaster 700. The difference was statistically significant (*t* = 2.509, *p* < 0.05) in the Pentacam Scheimpflug imaging. *p < 0.05.

In this study, about 53 eyes of 52 patients (25 female and 27 male) were evaluated. The mean age of the patients was 61.2 ± 4.9 years (range 53–76 years). According to the IOLMaster 700 lens images, the mean OD value measured with the image analysis software was 64.34 ± 23.31 and 63.81 ± 23.21 pixel units before and after mydriasis, respectively. There was no significant difference between the two groups (*t* = 0.455, *p* > 0.05). The mean PND value obtained from the Pentacam was 28.51 ± 11.42 and 25.41 ± 11.31 before and after mydriasis, respectively. On the contrary, the difference was statistically significant (*t* = 2.509, *p* < 0.05) (Figure 2).

**Figure 3:**
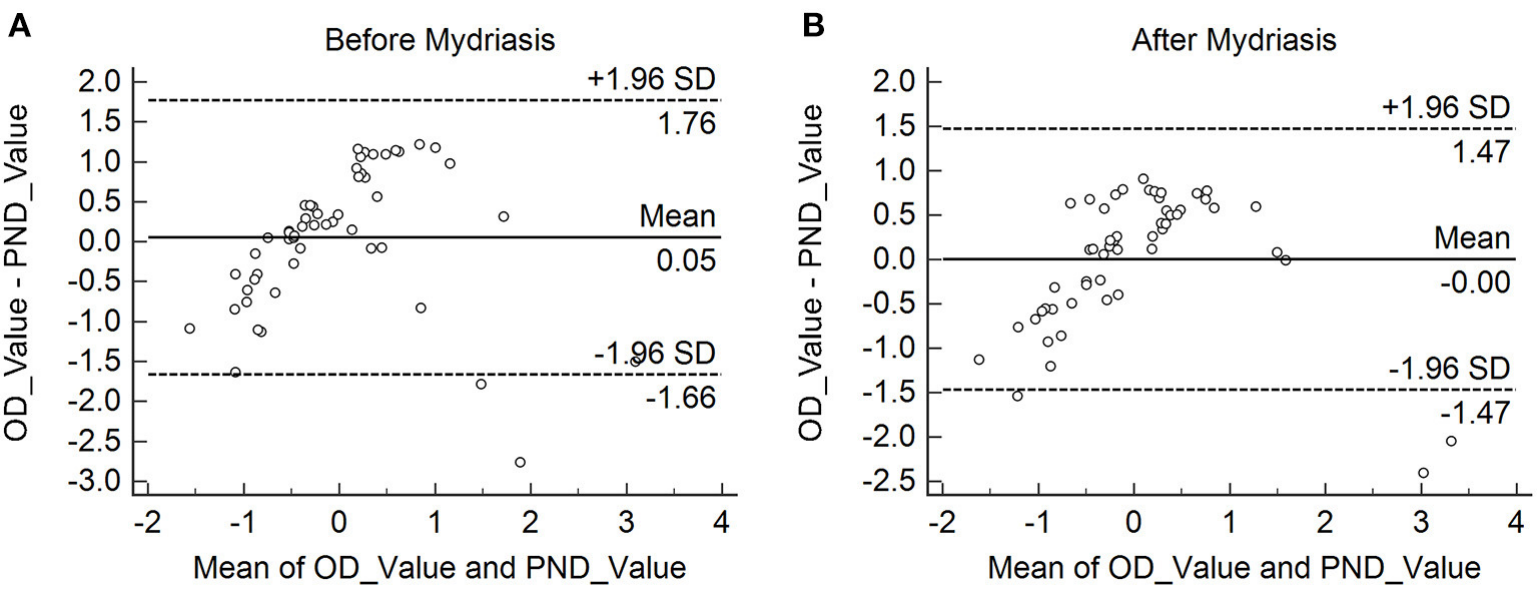
**(A,B)** The Bland–Altman plots for the comparisons between the values of OD and the Pentacam nucleus densitometry (PND) before and after mydriasis. The 95% limits of agreement (LoA) (95% uniformity bounds) were in the narrow range.

The Bland–Altman plots for the comparisons between the values of OD and PND were presented in Figure 3. The data were standardized owing to the discordance of the units of these two values. Lens nuclear density measured by the IOLMaster 700 and the Pentacam showed a high degree of consistency whether mydriasis is present or not. The 95% limits of agreement (LoA) (95% uniformity bounds) were in a narrow range as shown in Figure 3.

**Figure 4:**
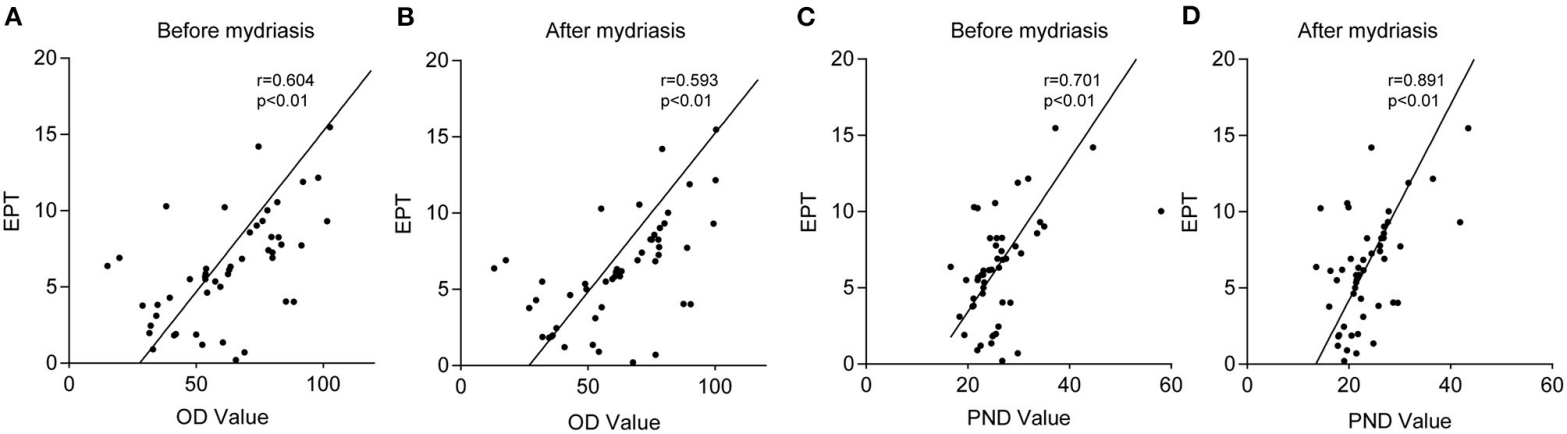
The correlations between the OD value and effective phacoemulsification time (EPT) value and the PND value and EPT value. Graphs **(A,B)** showing the correlation between the OD value and EPT value before and after mydriasis. Graph **(C,D)** showing the correlation between the PND value and EPT value before and after mydriasis. There was a positive correlation between the OD value and EPT value (*r* = 0.604 and *r* = 0.593, respectively, both *p* < 0.01) and between the PND value and EPT value (*r* = 0.701 and *r* = 0.891, respectively, both *p* < 0.01) before and after mydriasis.

The average EPT was 6.24 ± 3.49, there was a positive correlation between the OD value and EPT value (*r* = 0.604 and *r* = 0.593, respectively, both *p* < 0.01), and between the PND value and EPT value (*r* = 0.701 and *r* = 0.891, respectively, both *p* < 0.01) before and after mydriasis (Figure 4).

## Discussion

Nowadays, cataract is still the leading cause of blindness in the world and the risk factors of cataract increase with age. Lacking the objective measurements of the lens opacification is an obstacle to the diagnosis and prognosis of cataracts. The slit-lamp examination, based on the LOCSIII, is a common method to judge the density of the lens nucleus (18). However, it is very subjective because the results of multiple examinations may vary due to the different doctors (5).

Gupta et al. (19) reported in a similar study that there was a strong association between the lens nuclear density evaluated by the Pentacam Scheimpflug imaging and the LOCSIII grading. Edwards et al. (20) found that the lens cortex and nuclear density measured by the Pentacam possessed better repetition. In this study, six slices were selected from a global 360° scan by using the Pentacam rotating Scheimpflug imaging system for a mean value, therefore providing the more accurate measurements (21). However, the results obtained are limited by the peak density of the lens due to the method of linear measurement in the Pentacam Scheimpflug software. The Scheimpflug imaging shows the internal structure of the lens through the front refractive surface (i.e., the cornea and the front surface of the lens), which may distort the shape of the lens. This limitation is particularly evident in the different pupil sizes. In this study, the lens nuclear density is significantly different before and after mydriasis.

In contrast, the IOLMaster 700 based on SS-OCT can display the longitudinal structure of the eye and the overall image of the lens (22, 23), while SS-OCT uses a point detector to record the low coherence interference spectrum signal of the broadband scanning light source in time and achieves the parallel acquisition of the longitudinal results of the eye through the Fourier transform. Therefore, the IOLMaster 700 has better penetrability and repeatability. This study shows that owing to the different principles of the two instruments, the IOLMaster 700 measuring results with better penetration is not affected by the pupil size and there is no significant difference before and after mydriasis. This study also reported that the mean OD and PND values before mydriasis were higher compared to after mydriasis, though no statistical difference in the IOLMaster 700, which may be because more light can enter the eye after mydriasis, resulting in a slight decrease in the density of lens nucleus. In addition, some studies have pointed out that even if the pupil is completely dilated, the Pentacam still has an insufficient display of the posterior capsule of the lens (24, 25), while the IOLMaster 700 can clearly display the cornea, iris, lens, retina, and other intraocular structures. Hence, the IOLMaster 700 has more advantages in the measurement.

This study shows that both the IOLMaster 700 and the Pentacam are highly consistent in the measurement of the lens nuclear density regardless of mydriasis. Therefore, it is feasible to measure objectively and quantitatively the degree of cataract lens opacity by the IOLMaster 700. Moreover, the lens density acquired by the IOLMaster 700 without being affected by the pupil size is more preoperative instructive for some patients who cannot achieve mydriasis such as glaucoma, iris synechia, and other eye diseases. More accurate assessment on the lens opacity degree with the IOLMaster 700 is helpful to avoid the introperative and postoperative complications of phacoemulsification in the cataract population with the above situation. However, the limitation should be noted in this study: the OD value of the IOLMaster 700 is obtained by the image analysis software and it is based on the analysis of image gray level. This method of measurement is too expensive to be used in clinical work, so the combination of the image analysis software with the automatic version and the IOLMaster 700 can greatly improve its practicability.

In addition, the intraoperative energy parameters of phacoemulsification are closely related to the prognosis of the patients after the operation. For example, if the intraoperative energy parameters are too large, the common postoperative complications such as corneal edema on the first day after the operation will be obvious and the postoperative satisfaction will be greatly reduced (26). The degree of lens opacification plays a decisive role in the intraoperative energy parameters. Hence, the preoperative objective quantitative measurement of lens opacification state can predict the intraoperative energy parameters of phacoemulsification and combined with the rich clinical experience of the operator to evaluate the difficulty of the operation, which is conducive to the preoperative explanation, so as to increase the satisfaction of the patients after the operation. Therefore, we explored the influence of lens nuclear density on the energy parameters of phacoemulsification and concluded that the OD value and PND value before and after mydriasis were positively correlated with the EPT, which was the same compared to most research studies (27, 28), i.e., the higher the density of lens nucleus, the stronger the energy required for phacoemulsification during the operation. One of the key factors for the prognosis of cataract surgery is the energy parameters of phacoemulsification during the operation (29, 30). Therefore, the density of the lens nucleus obtained by the IOLMaster 700 can be used to predict the energy and time required for the phacoemulsification during the operation (31), which may help to optimize the treatment plan and better visual effect.

In conclusion, the IOLMaster 700 based on SS-OCT combined with the image analysis software is a feasible technology to measure the lens nuclear density. The result is consistent with that of the Pentacam anterior segment analyzer, which is commonly used in the clinic. It shows that the IOLMaster 700 can reasonably assemble the image analysis software to measure the lens nucleus density instead of the Pentacam Scheimpflug imaging. The IOLMaster 700 can provide patients with cataracts with better vision and visual experience from three aspects: the quantitative analysis of cataract lens opacity before phacoemulsification, measurement of eye biological indicators (32, 33), and surgical treatment.

## Data Availability Statement

The raw data supporting the conclusions of this article will be made available by the authors, without undue reservation.

## Ethics Statement

The studies involving human participants were reviewed and approved by the Ethics Committee of the Fourth Affiliated Hospital of China Medical University. The patients/participants provided their written informed consent to participate in this study.

## Author Contributions

BL and MS contributed to the data collection, original idea, data analysis, manuscript writing, and revision. YL and YH provided technical support. All authors contributed to the article and approved the submitted version.

## Funding

This study was funded by Natural Science Foundation of Liaoning Province (Grant No. 2019-ZD-0746) and Scientific Research Funding for Education Department of Liaoning Province (Grant No. ZD2020002).

## Conflict of Interest

The authors declare that the research was conducted in the absence of any commercial or financial relationships that could be construed as a potential conflict of interest.

## Publisher's Note

All claims expressed in this article are solely those of the authors and do not necessarily represent those of their affiliated organizations, or those of the publisher, the editors and the reviewers. Any product that may be evaluated in this article, or claim that may be made by its manufacturer, is not guaranteed or endorsed by the publisher.

## References

[1] Resnikoff S Pascolini D Etya'ale D Kocur I Pararajasegaram R Pokharel GP . Global data on visual impairment in the year 2002. Bull World Health Organ. (2004) 82:844–51. 10.1590/S0042-96862004001100009 15640920 PMC2623053

[2] Chylack LT Jr Wolfe JK Singer DM Leske MC Bullimore MA Bailey IL . The lens opacities classification system III. The longitudinal study of Cataract Study Group. Arch Ophthalmol. (1993) 111:831-6. 10.1001/archopht.1993.01090060119035 8512486

[3] Faria-Correia F Ramos I Lopes B Monteiro T Franqueira N Ambrósio R Jr. Comparison of dysfunctional lens index and Scheimpflug lens densitometry in the evaluation of age-related nuclear cataracts. J Refract Surg. (2016) 32:244-8. 10.3928/1081597X-20160209-01 27070231

[4] Zarnowski T Rejdak R Zielinska-Rzecka E Zrenner E Grieb P Zagórski Z . Elevated concentrations of kynurenic acid, a tryptophan derivative, in dense nuclear cataracts. Curr Eye Res. (2007) 32:27–32. 10.1080/02713680601090965 17364732

[5] Grewal DS Brar GS Grewal SP. Correlation of nuclear cataract lens density using Scheimpflug images with Lens Opacities Classification System III and visual function. Ophthalmology. (2009) 116:1436–43. 10.1016/j.ophtha.2009.03.002 19500847

[6] Meduri A Urso M Signorino GA Rechichi M Mazzotta C Kaufman S. Cataract surgery on post radial keratotomy patients. Int J Ophthalmol. (2017) 10:1168–70. 10.18240/ijo.2017.07.23 28730124 PMC5514283

[7] Faria-Correia F Lopes B Monteiro T Franqueira N AmbrKaufman S. Cataract surgery on post radial keratotomy pafront aberrations in patients with mild nuclear cataract. J Cataract Refract Surg. (2016) 42:405-11. 10.1016/j.jcrs.2015.10.069 27063521

[8] Artal P Benito A P.rgs with mild nuclear cataract. mbrK, et al. An objective scatter index based on double-pass retinal images of a point source to classify cataracts. PLoS ONE. (2011) 6:e16823. 10.1371/journal.pone.0016823 21326868 PMC3033912

[9] Faria-Correia F Lopes B Monteiro T Franqueira N Ambrl images of a point source to classify cataracts. omy patients. Classification System III and visual function. f age-r time in mild nuclear cataracts. Int Ophthalmol. (2018) 38:1103-1110. 10.1007/s10792-017-0566-7 28550347

[10] Lim SA Hwang J Hwang KY Chung SH. Objective assessment of nuclear cataract: comparison of double-pass and Scheimpflug systems. J Cataract Refract Surg. (2014) 40:716-21. 10.1016/j.jcrs.2013.10.032 24767907

[11] De Bernardo M Borrelli M Imparato R Cione F Rosa N. Anterior chamber depth measurement before and after photorefractive keratectomy. Comparison between IOLMaster and Pentacam. Photodiagnosis Photodyn Ther. (2020) 32:101976. 10.1016/j.pdpdt.2020.101976 32841750

[12] Salouti R Kamalipour A Masihpour N . Effect of photorefractive keratectomy on agreement of anterior segment variables obtained by a swept-source biometer vs a Scheimpflug-based tomographer. J Cataract Refract Surg. (2020) 46:1229–235. 10.1097/j.jcrs.0000000000000252 32483074

[13] Pei X Bao Y Chen Y Li X. Correlation of lens density measured using the Pentacam Scheimpflug system with the Lens Opacities Classification System III grading score and visual acuity in age-related nuclear cataract. Br J Ophthalmol. (2008) 92:1471–75. 10.1136/bjo.2007.136978 18586899

[14] Patr788h 10.molhen Y, Li X. Correlation of lens density measured using the Pentacam Scheimpflug system with the Lens Opacities Classification System III grading score and visual acuity in age-relate*Eur J Ophthalmol*. (2013) 23:789-792. 10.5301/ejo.5000293 18586899

[15] Potsaid B Baumann B Huang D Barry S Cable AE Schuman JS . Ultrahigh speed 1050nm swept source/Fourier domain OCT retinal and anterior segment imaging at 100,000 to 400,000 axial scans per second. Opt Express. (2010) 18:20029–20048. 10.1364/OE.18.020029 20940894 PMC3136869

[16] Wojtkowski M Srinivasan V Fujimoto JG Ko T Schuman JS Kowalczyk A . Three-dimensional retinal imaging with high-speed ultrahigh-resolution optical coherence tomography. Ophthalmology. (2005) 112:1734–1746. 10.1016/j.ophtha.2005.05.023 16140383 PMC1939719

[17] Wojtkowski M Srinivasan V Fujimoto JG . Three-dimensional retinal imaging with high-speed ultrahigh-resolution optical coherence tomography. J Cataract Refract Surg. (2018) 44:1478–81. 10.1016/j.jcrs.2018.08.009 16140383 PMC1939719

[18] Gali HE Sella R Afshari NA. Cataract grading systems: a review of past and present. Curr Opin Ophthalmol. (2019) 30:13-18. 10.1097/ICU.0000000000000542 30489359

[19] Gupta M Ram J Jain A Sukhija J Chaudhary M. Correlation of nuclear density using the Lens Opacity Classification System III versus Scheimpflug imaging with phacoemulsification parameters. J Cataract Refract Surg. (2013) 39:1818–23. 10.1016/j.jcrs.2013.05.052 24286839

[20] Edwards PA Datiles MB Green SB. Reproducibility study on the scheimpflug cataract video camera. Curr Eye Res. (1988) 7:955-60. 10.3109/02713688809015140 3229123

[21] Nixon DR. Preoperative cataract grading by Scheimpflug imaging and effect on operative fluidics and phacoemulsification energy. J Cataract Refract Surg. (2010) 36:242-6. 10.1016/j.jcrs.2009.08.032 20152604

[22] Panthier C Burgos J Rouger H Saad A Gatinel D. New objective lens density quantification method using swept-source optical coherence tomography technology: Comparison with existing methods. J Cataract Refract Surg. (2017) 43:1575–1581. 10.1016/j.jcrs.2017.09.028 29335103

[23] Kim YN Park JH Tchah H. Quantitative analysis of lens nuclear density using Optical Coherence Tomography (OCT) with a Liquid Optics Interface: Correlation between OCT Images and LOCS III Grading. J Ophthalmol. (2016) 2016:3025413. 10.1155/2016/3025413 27651952 PMC5019885

[24] Garza-Leon M. Fuentes-de la Fuente HA, GarcGrading. ens nuRepeatability of ocular biometry with IOLMaster700 in subjects with clear lens. Int Ophthalmol. (2017) 37:1133–1138. 10.1007/s10792-016-0380-7 27770390

[25] Dubbelman M Van der Heijde GL. The shape of the aging human lens: curvature, equivalent refractive index and the lens paradox. Vision Res. (2001) 41:1867-77. 10.1016/s0042-6989(01)00057-8 11369049

[26] Kim JS Chung SH Joo CK. Clinical application of a Scheimpflug system for lens density measurements in phacoemulsification. Erratum in: J Cataract Refract Surg. (2009) 35:1483. 10.1016/j.jcrs.2009.02.032 19545809

[27] Davison JA Chylack LT. Clinical application of the lens opacities classification system III in the performance of phacoemulsification. J Cataract Refract Surg. (2003) 29:138-45. 10.1016/s0886-3350(02)01839-4 12551681

[28] Tanaka T Koshika S Usui M. Cataract surgery using the bimanual phacoemulsification technique with an Accurus system and Mackool microphaco tip. J Cataract Refract Surg. (2007) 33:1770-4. 10.1016/j.jcrs.2007.06.021 17889775

[29] Rekas M Montés-Micó R Krix-Jachym K Kluś A Stankiewicz A Ferrer-Blasco T. Comparison of torsional and longitudinal modes using phacoemulsification parameters. J Cataract Refract Surg. (2009) 35:1719-24. 10.1016/j.jcrs.2009.04.047 19781466

[30] Fine IH Packer M Hoffman RS. Power modulations in new phacoemulsification technology: improved outcomes. J Cataract Refract Surg. (2004) 30:1014-9. 10.1016/j.jcrs.2003.09.062 15130637

[31] Lim SA Shin JY Chung SH. Useful prediction of phacodynamics by scheimpflug lens densitometry in patients over age 70. Semin Ophthalmol. (2017) 32:482–7. 10.3109/08820538.2015.1120756 27092580

[32] Montés-Micó R. Evaluation of six biometers based on different optical technologies. J Cataract Refract Surg. (2021). 10.1097/j.jcrs.0000000000000690. [Epub ahead of print]. 34091551 PMC8700306

[33] Chirapapaisan C Srivannaboon S Chonpimai P. Efficacy of swept-source optical coherence tomography in axial length measurement for advanced cataract patients. Optom Vis Sci. (2020) 97:186-191. 10.1097/OPX.0000000000001491 32168241

